# Co-infection with Avian (H7N9) and Pandemic (H1N1) 2009 Influenza Viruses, China

**DOI:** 10.3201/eid2104.141560

**Published:** 2015-04

**Authors:** Wanju Zhang, Dongyi Zhu, Di Tian, Lei Xu, Zhaokui Zhu, Zheng Teng, Jing He, Shan Shan, Yi Liu, Wei Wang, Zhenghong Yuan, Tao Ren, Yunwen Hu

**Affiliations:** Shanghai Public Health Clinical Center of Fudan University, Shanghai, China. (W. Zhang, D. Tian, L. Xu, J. He, Y. Liu, W. Wang, Z. Yuan, Y. Hu);; East Hospital, Tongji University School of Medicine, Shanghai (D. Zhu, S. Shan, T. Ren);; Shanghai Municipal Center for Disease Control and Prevention, Shanghai (Z. Zhu, Z. Teng);; Shanghai Medical College of Fudan University, Shanghai (Z. Yuan, Y. Hu)

**Keywords:** co-infection, H7N9, H1N1, A(H1N1)pdm09, influenza, viruses, China, pandemic

**To the Editor:** Since the first case of avian-origin influenza (H7N9) virus infection was reported in March 2013 in Shanghai, China, this virus has caused ≈400 confirmed cases (http://www.who.int/influenza/human_animal_interface/influenza_h7n9/Data_Reports/en/). According to the Chinese Influenza Weekly Report (http://www.cnic.org.cn/eng/show.php?contentid=699), during the winter of 2013–14, multiple influenza viruses that infect humans co-circulated in southern China; these viruses were influenza A(H1N1)pdm09, seasonal influenza A(H3N2), and influenza B viruses, together with avian influenza (H7N9) virus,. Co-infection of humans with avian virus subtype H7N9 and human virus subtypes were reported in Jiangsu Province in June 2013 (*1*) and in Zhejiang Province in January 2014 (*2*), respectively. We report a fatal case of co-infection with influenza (H7N9) and A(H1N1)pdm09 viruses, which was further confirmed by virus isolation and neutralization antibody detection. 

In a retrospective study, we identified co-infection with A(H1N1)pdm09 and novel avian-origin H7N9 virus in a 61-year-old female patient from the urban area of Shanghai, China. She had a 3-year history of thrombocythemia. In October 2013, she received an annual vaccine that included an A/California/7/2009(H1N1)pdm09–like virus, an A/Texas/50/2012(H3N2)–like virus, and an influenza B virus, as recommended by the World Health Organization (http://www.who.int/influenza/vaccines/virus/recommendations/2013_14_north/en/). On December 26, 2013, the patient experienced influenza-like symptoms; the next day, she was hospitalized. Despite oral administration of oseltamivir, a rapidly progressive pneumonia developed, and the patient died of multiple organ failure 9 days after admission. On December 31, 2013 (day 5), 1 serum and 2 throat swab samples were collected; both throat swab samples were positive for influenza A and A(H1N1)pdm09 virus, according to the universal and subtype-specific primers provided by the Chinese Center for Disease Control and Prevention (http://www.who.int/influenza/resources/documents/diagnostic_recommendations/en/). On January 3, 2014 (day 8), 1 bronchial secretion and 1 serum sample were also collected.

Recent DNA sequencing analysis of the stored bronchial secretion specimen, performed on a Roche 454 platform (*3*) (Roche, Basel, Switzerland) showed co-existence of H1, H7, N1, and N9 genomic segments, which were further confirmed by real-time reverse transcription PCR (RT-PCR). The cycle threshold values of H7 and H1 were 35.65 and 29.94, respectively.

We then determined antibody levels of the 2 serum samples collected on days 5 and 8 by using a microneutralization assay (*4*) with MDCK cells infected with 2 representative strains, A/Shanghai/4664T/2013(H7N9) and A/Shanghai/37T/2009(H1N1), respectively. The samples were serially diluted 2-fold (1:10 to 1:1,280) at a starting dilution of 1:10; titers were expressed as the reciprocal of the highest dilution of 50% neutralization. The microneutralization titers against novel subtype H7N9 virus were 160 and 320 for the samples collected on days 5 and 8, respectively. This finding indicated that the patient could have been infected by novel subtype H7N9 viruses (*5*); microneutralization titers against A/Shanghai/37T/2009(H1N1) were 320 for both samples.

To determine whether the reassortment had occurred between the 2 subtypes of influenza A viruses during the co-infection, we isolated the virus strains from the bronchial secretion on either MDCK cells (A[H1N1]pdm09) (http://www.who.int/influenza/gisrs_laboratory/manual_diagnosis_surveillance_influenza/en/) or embryonated eggs (H7N9) through several passages. The hemagglutination titers of the isolated H7N9 (A/Shanghai/Mix1/2014[H7N9]) and A(H1N1)pdm09 viruses (A/Shanghai/Mix1/2014[H1N1]) in allantoic fluid were 32 and 8, respectively. Cultures of the 2 isolates were subjected to RT-PCR for detection of 8 genomic segments. The RT-PCR products were sequenced on an ABI 3730XL Automatic DNA Analyzer by using an ABI Prism BigDye Terminator v3.1 Cycle Sequencing Kit (both from Applied Biosystems, Foster City, CA, USA). The full-genome sequences were submitted to the National Center for Biotechnology Information Influenza Virus Resource (GenBank accession nos. KJ946415, KJ946416 and KP058484–KP058489 for A/Shanghai/Mix1/201[H7N9] and KM607860, KM607861 and KP058490–KP058495 for A/Shanghai/Mix1/2014[H1N1]).

Phylogenetic trees were constructed by using the neighbor-joining method in MEGA 5.1 (http://www.megasoftware.net) to estimate relationships with selected influenza A virus reference sequences obtained from GenBank and the Global Initiative on Sharing Avian Influenza Data database. DNA sequence analysis showed that no genetic reassortment had occurred between the 2 subtypes because only H7N9 or A(H1N1)pdm09 genomic fragments were found in the culture of the respective isolate (Figure; Technical Appendix). Phylogenetic analyses of the hemagglutinin and neuraminidase genes revealed that the A/Shanghai/Mix1/2014(H7N9) and A/Shanghai/Mix1/2014(H1N1) viruses were clustered into the clade of A/Shanghai/01/2014(H7N9)-like viruses (*6*) (Figure, panels A and B) and A/Shanghai/6109/2014(H1N1)-like viruses (Figure, panels C and D), respectively. In addition, the phylogenetic trees of the 6 internal genes were close to those of H7N9 viruses or A(H1N1)pdm09-lineage viruses that had recently circulated in China (Technical Appendix).

**Figure:**
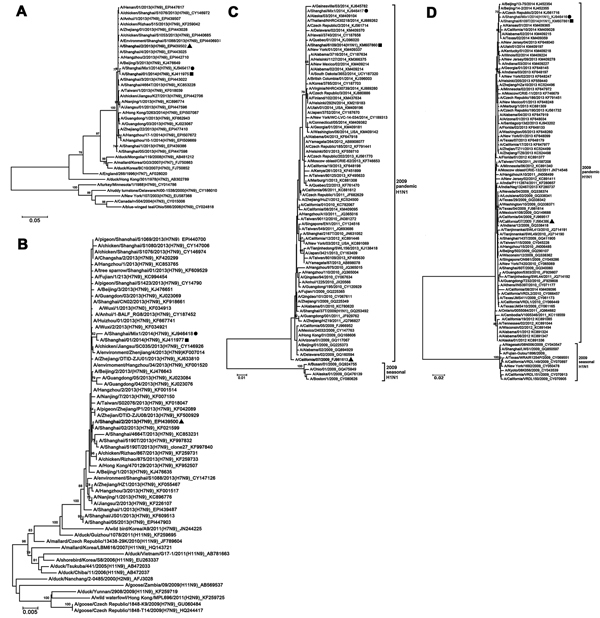
Phylogenetic analyses of A/Shanghai/Mix1/2014(H7N9) and A/Shanghai/Mix1/2014(H1N1) viruses from Shanghai, China, January 2014. A) Hemagglutinin and B) neuraminidase of A/Shanghai/Mix1/2014(H7N9); C) hemagglutinin and D) neuraminidase of A/Shanghai/Mix1/2014(H1N1). Multiple alignments were constructed by using the ClustalW (http://www.clustal.org/) algorithm based on the sequences of the hemagglutinin and neuraminidase genes. Genetic distances were calculated by using the Kimura 2-parameter method, and phylogenetic trees were constructed by using the neighbor-joining method with bootstrap analysis of 1,000 replicates in MEGA 5.1 (http://www.megasoftware.net). Horizontal distances are proportional to genetic distances. Numbers next to nodes indicate bootstrap value percentages (>70%). Circles indicate the A/Shanghai/Mix1/2014(H7N9) and A/Shanghai/Mix1/2014(H1N1) virus strains isolated from the co-infection patient from Shanghai, China. Triangles indicate the representative strains of the Shanghai area during the same period as the influenza (H7N9) outbreak in China. Squares indicate the representative strains of the Shanghai area during the same wave of the H7N9 outbreak in China. Scale bars indicate nucleotide substitutions per site. The Global Initiative on Sharing Avian Influenza Data database or GenBank accession numbers of the influenza viruses used in the construction of the phylogenetic analyses are displayed in the phylogenetic trees, which follow the taxon names.

Co-infections with H7N9 and other influenza subtypes have been reported from Jiangsu (*1*) and Zhejiang (*2*) Provinces as well as in our study, indicating the potential risk for co-infection during the influenza season in China. Although reassortment was not detected in this co-infection, a potential risk for emergence of a new pandemic strain by reassortment between these 2 viruses (with humans as mixing vessels) should not be ignored. To reduce the risk for emergence of new viral subtypes, the public health and scientific communities should enhance surveillance for co-infection with influenza (H7N9) virus and other influenza virus subtypes.

Technical Appendix Phylogenetic analysis of 12 internal genes of the co-infection viruses in Shanghai, China.
