# Supplementary material for: Co-infection with Avian (H7N9) and Pandemic (H1N1) 2009 Influenza Viruses, China
Source: Emerg Infect Dis. 2015 Apr;21(4):715–8. doi: 10.3201/eid2104.141560 (PMC4378472; doi:10.3201/eid2104.141560)
Supplement: Technical Appendix — Phylogenetic analysis of 12 internal genes of the co-infection viruses in Shanghai, China. [file 14-1560-Techapp-s1.pdf]

Article DOI: http://dx.doi.org/10.3201/eid2104.141560

Co-infection with Avian (H7N9) and Pandemic (H1N1) 2009 Influenza Viruses, China

Technical Appendix

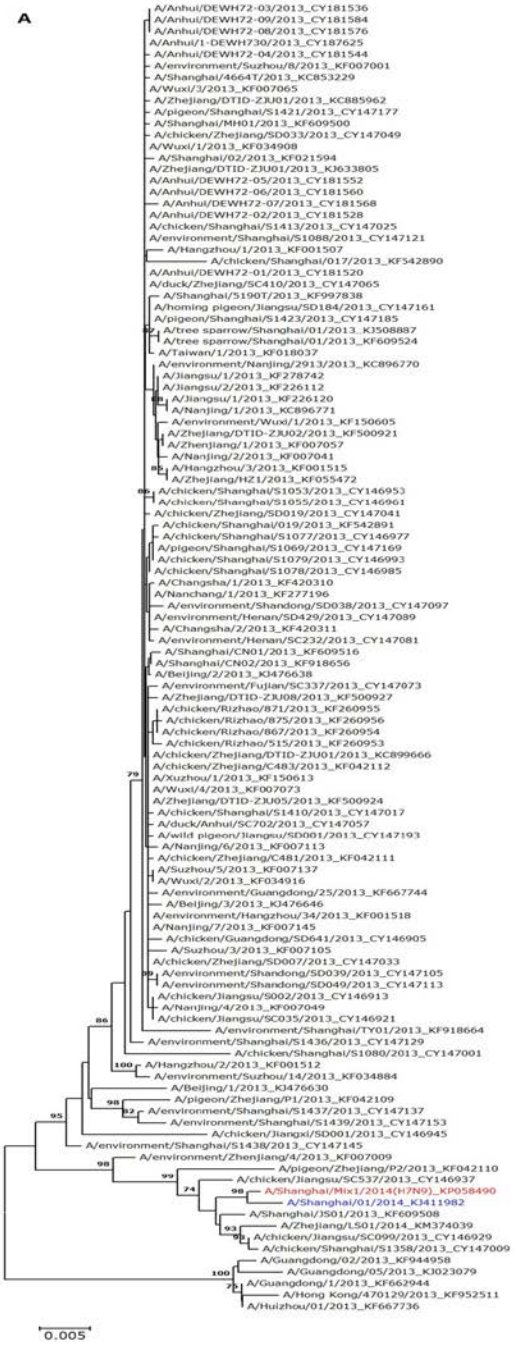

B

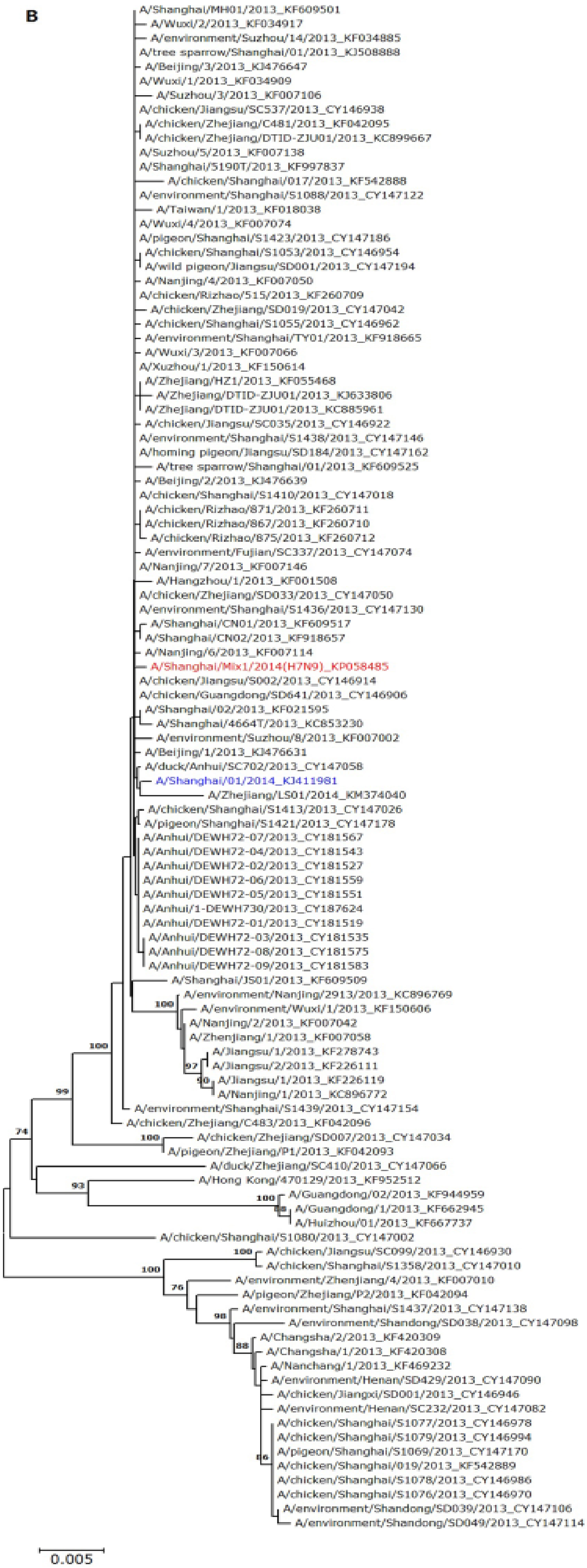
A/Shanghai/MH01/2013_KF609501
A/Wuxi/2/2013_KF034917
A/environment/Suzhou/14/2013_KF034885
A/tree sparrow/Shanghai/01/2013_KJ508888
A/Beijing/3/2013_KJ476647
A/Wuxi/1/2013_KF034909
A/Suzhou/3/2013_KF007106
A/chicken/Jiangsu/SC537/2013_CY146938
A/chicken/Zhejiang/C481/2013_KF042095
A/chicken/Zhejiang/DTID-ZJU01/2013_KC899667
A/Suzhou/5/2013_KF007138
A/Shanghai/5190T/2013_KF997837
A/chicken/Shanghai/017/2013_KF542888
A/environment/Shanghai/S1088/2013_CY147122
A/Taiwan/1/2013_KF018038
A/Wuxi/4/2013_KF007074
A/pigeon/Shanghai/S1423/2013_CY147186
A/chicken/Shanghai/S1053/2013_CY146954
A/wild pigeon/Jiangsu/SD001/2013_CY147194
A/Nanjing/4/2013_KF007050
A/chicken/Rizhao/515/2013_KF260709
A/chicken/Zhejiang/SD019/2013_CY147042
A/chicken/Shanghai/S1055/2013_CY146962
A/environment/Shanghai/TY01/2013_KF918665
A/Wuxi/3/2013_KF007066
A/Xuzhou/1/2013_KF150614
A/Zhejiang/HZ1/2013_KF055468
A/Zhejiang/DTID-ZJU01/2013_KJ633806
A/Zhejiang/DTID-ZJU01/2013_KC885961
A/chicken/Jiangsu/SC035/2013_CY146922
A/environment/Shanghai/S1438/2013_CY147146
A/homing pigeon/Jiangsu/SD184/2013_CY147162
A/tree sparrow/Shanghai/01/2013_KF609525
A/Beijing/2/2013_KJ476639
A/chicken/Shanghai/S1410/2013_CY147018
A/chicken/Rizhao/871/2013_KF260711
A/chicken/Rizhao/867/2013_KF260710
A/chicken/Rizhao/875/2013_KF260712
A/environment/Fujian/SC337/2013_CY147074
A/Nanjing/7/2013_KF007146
A/Hangzhou/1/2013_KF001508
A/chicken/Zhejiang/SD033/2013_CY147050
A/environment/Shanghai/S1436/2013_CY147130
A/Shanghai/CN01/2013_KF609517
A/Shanghai/CN02/2013_KF918657
A/Nanjing/6/2013_KF007114
A/Shanghai/Mix1/2014(H7N9)_KP058485
A/chicken/Jiangsu/SD002/2013_CY146914
A/chicken/Guangdong/SD641/2013_CY146906
A/Shanghai/02/2013_KF021595
A/Shanghai/4664T/2013_KC853230
A/environment/Suzhou/8/2013_KF007002
A/Beijing/1/2013_KJ476631
A/duck/Anhui/SC702/2013_CY147058
A/Shanghai/01/2014_KJ411981
A/Zhejiang/LS01/2014_KM374040
A/chicken/Shanghai/S1413/2013_CY147026
A/pigeon/Shanghai/S1421/2013_CY147178
A/Anhui/DEWH72-07/2013_CY181567
A/Anhui/DEWH72-04/2013_CY181543
A/Anhui/DEWH72-02/2013_CY181527
A/Anhui/DEWH72-06/2013_CY181559
A/Anhui/DEWH72-05/2013_CY181551
A/Anhui/1-DEWH730/2013_CY187624
A/Anhui/DEWH72-01/2013_CY181519
A/Anhui/DEWH72-03/2013_CY181535
A/Anhui/DEWH72-08/2013_CY181575
A/Anhui/DEWH72-09/2013_CY181583
A/Shanghai/JS01/2013_KF609509
A/environment/Nanjing/2913/2013_KC896769
100
A/environment/Wuxi/1/2013_KF150606
A/Nanjing/2/2013_KF007042
A/Zhenjiang/1/2013_KF007058
A/Jiangsu/1/2013_KF278743
97
A/Jiangsu/2/2013_KF226111
A/Jiangsu/1/2013_KF226119
A/Nanjing/1/2013_KC896772
100
99
A/environment/Shanghai/S1439/2013_CY147154
A/chicken/Zhejiang/C483/2013_KF042096
74
100
A/chicken/Zhejiang/SD007/2013_CY147034
A/pigeon/Zhejiang/P1/2013_KF042093
A/duck/Zhejiang/SC410/2013_CY147066
A/Hong Kong/470129/2013_KF952512
93
100
A/Guangdong/02/2013_KF944959
A/Guangdong/1/2013_KF662945
A/Huizhou/01/2013_KF667737
A/chicken/Shanghai/S1080/2013_CY147002
100
A/chicken/Jiangsu/SC099/2013_CY146930
A/chicken/Shanghai/S1358/2013_CY147010
100
76
A/environment/Zhenjiang/4/2013_KF007010
A/pigeon/Zhejiang/P2/2013_KF042094
A/environment/Shanghai/S1437/2013_CY147138
98
A/environment/Shandong/SD038/2013_CY147098
A/Changsha/2/2013_KF420309
88
A/Changsha/1/2013_KF420308
A/Nanchang/1/2013_KF469232
A/environment/Henan/SD429/2013_CY147090
A/chicken/Jiangxi/SD001/2013_CY146946
A/environment/Henan/SC232/2013_CY147082
A/chicken/Shanghai/S1077/2013_CY146978
A/chicken/Shanghai/S1079/2013_CY146994
A/pigeon/Shanghai/S1069/2013_CY147170
A/chicken/Shanghai/019/2013_KF542889
A/chicken/Shanghai/S1078/2013_CY146986
A/chicken/Shanghai/S1076/2013_CY146970
A/environment/Shandong/SD039/2013_CY147106
A/environment/Shandong/SD049/2013_CY147114
0.005

C

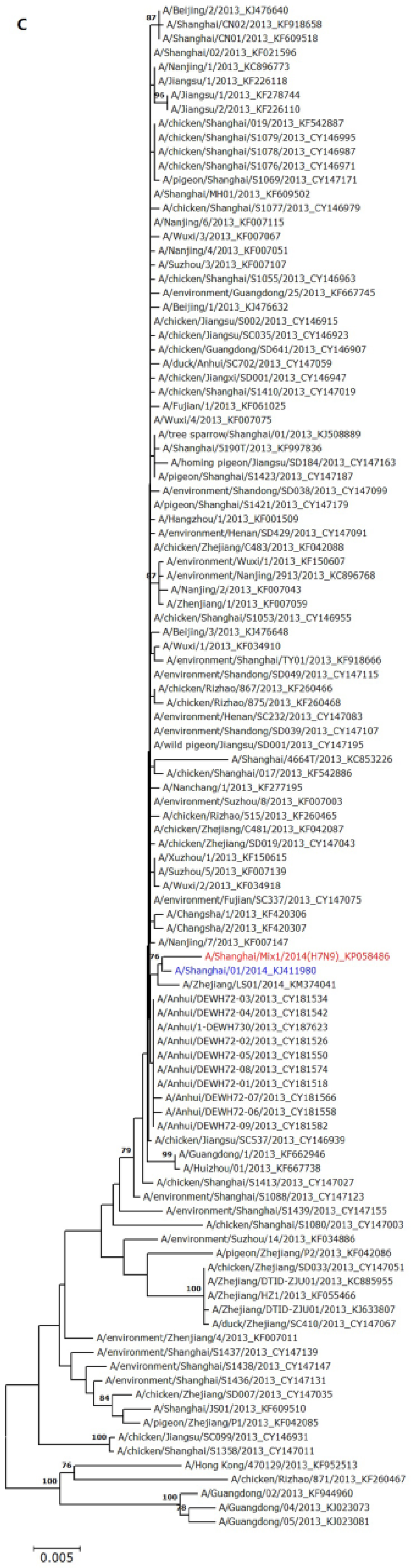

87
A/Beijing/2/2013_KJ476640
A/Shanghai/CN02/2013_KF918658
A/Shanghai/CN01/2013_KF609518
A/Shanghai/02/2013_KF021596
A/Nanjing/1/2013_KC896773
A/Jiangsu/1/2013_KF226118
96
A/Jiangsu/1/2013_KF278744
A/Jiangsu/2/2013_KF226110
A/chicken/Shanghai/019/2013_KF542887
A/chicken/Shanghai/S1079/2013_CY146995
A/chicken/Shanghai/S1078/2013_CY146987
A/chicken/Shanghai/S1076/2013_CY146971
A/pigeon/Shanghai/S1069/2013_CY147171
A/Shanghai/MH01/2013_KF609502
A/chicken/Shanghai/S1077/2013_CY146979
A/Nanjing/6/2013_KF007115
A/Wuxi/3/2013_KF007067
A/Nanjing/4/2013_KF007051
A/Suzhou/3/2013_KF007107
A/chicken/Shanghai/S1055/2013_CY146963
A/environment/Guangdong/25/2013_KF667745
A/Beijing/1/2013_KJ476632
A/chicken/Jiangsu/S002/2013_CY146915
A/chicken/Jiangsu/SC035/2013_CY146923
A/chicken/Guangdong/SD641/2013_CY146907
A/duck/Anhui/SC702/2013_CY147059
A/chicken/Jiangxi/SD001/2013_CY146947
A/chicken/Shanghai/S1410/2013_CY147019
A/Fujian/1/2013_KF061025
A/Wuxi/4/2013_KF007075
A/tree sparrow/Shanghai/01/2013_KJ508889
A/Shanghai/5190T/2013_KF997836
A/homing pigeon/Jiangsu/SD184/2013_CY147163
A/pigeon/Shanghai/S1423/2013_CY147187
A/environment/Shandong/SD038/2013_CY147099
A/pigeon/Shanghai/S1421/2013_CY147179
A/Hangzhou/1/2013_KF001509
A/environment/Henan/SD429/2013_CY147091
A/chicken/Zhejiang/C483/2013_KF042088
A/environment/Wuxi/1/2013_KF150607
87
A/environment/Nanjing/2913/2013_KC896768
A/Nanjing/2/2013_KF007043
A/Zhenjiang/1/2013_KF007059
A/chicken/Shanghai/S1053/2013_CY146955
A/Beijing/3/2013_KJ476648
A/Wuxi/1/2013_KF034910
A/environment/Shanghai/TY01/2013_KF918666
A/environment/Shandong/SD049/2013_CY147115
A/chicken/Rizhao/867/2013_KF260466
A/chicken/Rizhao/875/2013_KF260468
A/environment/Henan/SC232/2013_CY147083
A/environment/Shandong/SD039/2013_CY147107
A/wild pigeon/Jiangsu/SD001/2013_CY147195
A/Shanghai/4664T/2013_KC853226
A/chicken/Shanghai/017/2013_KF542886
A/Nanchang/1/2013_KF277195
A/environment/Suzhou/8/2013_KF007003
A/chicken/Rizhao/515/2013_KF260465
A/chicken/Zhejiang/C481/2013_KF042087
A/chicken/Zhejiang/SD019/2013_CY147043
A/Xuzhou/1/2013_KF150615
A/Suzhou/5/2013_KF007139
A/Wuxi/2/2013_KF034918
A/environment/Fujian/SC337/2013_CY147075
A/Changsha/1/2013_KF420306
A/Changsha/2/2013_KF420307
A/Nanjing/7/2013_KF007147
76
A/Shanghai/Mix1/2014(H7N9)_KP058486
A/Shanghai/01/2014_KJ411980
A/Zhejiang/LS01/2014_KM374041
A/Anhui/DEWH72-03/2013_CY181534
A/Anhui/DEWH72-04/2013_CY181542
A/Anhui/1-DEWH730/2013_CY187623
A/Anhui/DEWH72-02/2013_CY181526
A/Anhui/DEWH72-05/2013_CY181550
A/Anhui/DEWH72-08/2013_CY181574
A/Anhui/DEWH72-01/2013_CY181518
A/Anhui/DEWH72-07/2013_CY181566
A/Anhui/DEWH72-06/2013_CY181558
A/Anhui/DEWH72-09/2013_CY181582
A/chicken/Jiangsu/SC537/2013_CY146939
79
99
A/Guangdong/1/2013_KF662946
A/Huizhou/01/2013_KF667738
A/chicken/Shanghai/S1413/2013_CY147027
A/environment/Shanghai/S1088/2013_CY147123
A/environment/Shanghai/S1439/2013_CY147155
A/chicken/Shanghai/S1080/2013_CY147003
A/environment/Suzhou/14/2013_KF034886
A/pigeon/Zhejiang/P2/2013_KF042086
A/chicken/Zhejiang/SD033/2013_CY147051
A/Zhejiang/DTID-ZJU01/2013_KC885955
100
A/Zhejiang/HZ1/2013_KF055466
A/Zhejiang/DTID-ZJU01/2013_KJ633807
A/duck/Zhejiang/SC410/2013_CY147067
A/environment/Zhenjiang/4/2013_KF007011
A/environment/Shanghai/S1437/2013_CY147139
A/environment/Shanghai/S1438/2013_CY147147
A/environment/Shanghai/S1436/2013_CY147131
84
A/chicken/Zhejiang/SD007/2013_CY147035
A/Shanghai/JS01/2013_KF609510
A/pigeon/Zhejiang/P1/2013_KF042085
100
A/chicken/Jiangsu/SC099/2013_CY146931
A/chicken/Shanghai/S1358/2013_CY147011
76
A/Hong Kong/470129/2013_KF952513
100
A/chicken/Rizhao/871/2013_KF260467
100
A/Guangdong/02/2013_KF944960
A/Guangdong/04/2013_KJ023073
A/Guangdong/05/2013_KJ023081
0.005

D

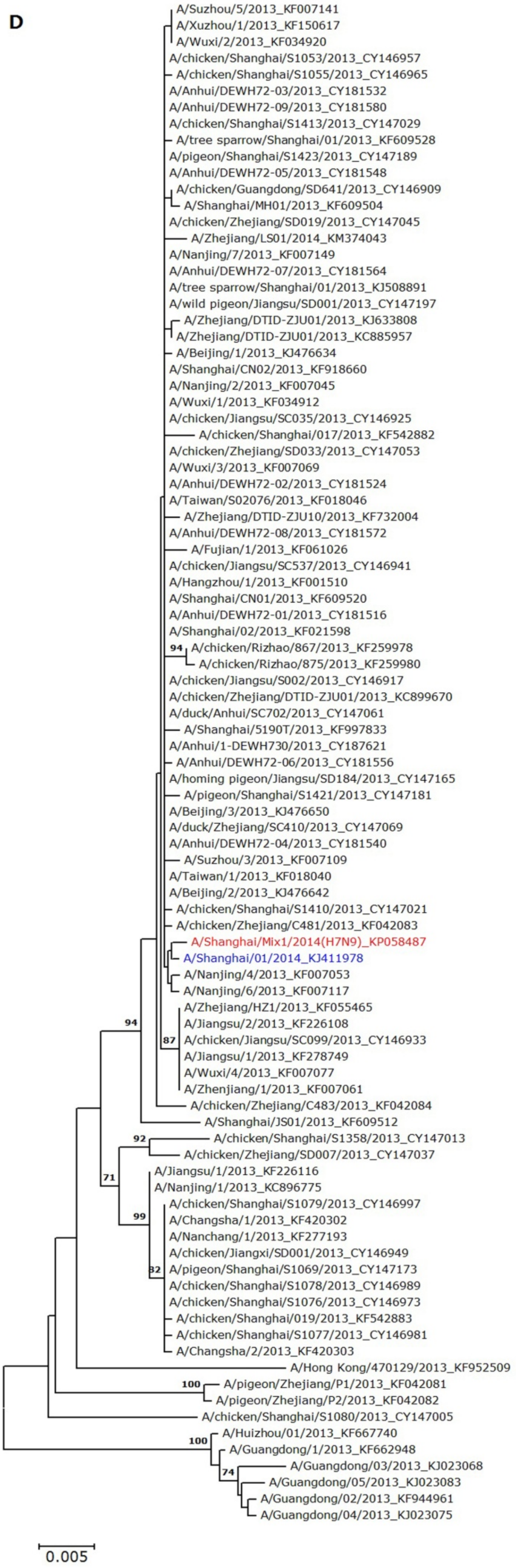
A/Suzhou/5/2013_KF007141
A/Xuzhou/1/2013_KF150617
A/Wuxi/2/2013_KF034920
A/chicken/Shanghai/S1053/2013_CY146957
A/chicken/Shanghai/S1055/2013_CY146965
A/Anhui/DEWH72-03/2013_CY181532
A/Anhui/DEWH72-09/2013_CY181580
A/chicken/Shanghai/S1413/2013_CY147029
A/tree sparrow/Shanghai/01/2013_KF609528
A/pigeon/Shanghai/S1423/2013_CY147189
A/Anhui/DEWH72-05/2013_CY181548
A/chicken/Guangdong/SD641/2013_CY146909
A/Shanghai/MH01/2013_KF609504
A/chicken/Zhejiang/SD019/2013_CY147045
A/Zhejiang/LS01/2014_KM374043
A/Nanjing/7/2013_KF007149
A/Anhui/DEWH72-07/2013_CY181564
A/tree sparrow/Shanghai/01/2013_KJ508891
A/wild pigeon/Jiangsu/SD001/2013_CY147197
A/Zhejiang/DTID-ZJU01/2013_KJ633808
A/Zhejiang/DTID-ZJU01/2013_KC885957
A/Beijing/1/2013_KJ476634
A/Shanghai/CN02/2013_KF918660
A/Nanjing/2/2013_KF007045
A/Wuxi/1/2013_KF034912
A/chicken/Jiangsu/SC035/2013_CY146925
A/chicken/Shanghai/017/2013_KF542882
A/chicken/Zhejiang/SD033/2013_CY147053
A/Wuxi/3/2013_KF007069
A/Anhui/DEWH72-02/2013_CY181524
A/Taiwan/S02076/2013_KF018046
A/Zhejiang/DTID-ZJU10/2013_KF732004
A/Anhui/DEWH72-08/2013_CY181572
A/Fujian/1/2013_KF061026
A/chicken/Jiangsu/SC537/2013_CY146941
A/Hangzhou/1/2013_KF001510
A/Shanghai/CN01/2013_KF609520
A/Anhui/DEWH72-01/2013_CY181516
A/Shanghai/02/2013_KF021598
94
A/chicken/Rizhao/867/2013_KF259978
A/chicken/Rizhao/875/2013_KF259980
A/chicken/Jiangsu/S002/2013_CY146917
A/chicken/Zhejiang/DTID-ZJU01/2013_KC899670
A/duck/Anhui/SC702/2013_CY147061
A/Shanghai/5190T/2013_KF997833
A/Anhui/1-DEWH730/2013_CY187621
A/Anhui/DEWH72-06/2013_CY181556
A/homing pigeon/Jiangsu/SD184/2013_CY147165
A/pigeon/Shanghai/S1421/2013_CY147181
A/Beijing/3/2013_KJ476650
A/duck/Zhejiang/SC410/2013_CY147069
A/Anhui/DEWH72-04/2013_CY181540
A/Suzhou/3/2013_KF007109
A/Taiwan/1/2013_KF018040
A/Beijing/2/2013_KJ476642
A/chicken/Shanghai/S1410/2013_CY147021
A/chicken/Zhejiang/C481/2013_KF042083
A/Shanghai/Mix1/2014(H7N9)_KP058487
A/Shanghai/01/2014_KJ411978
A/Nanjing/4/2013_KF007053
A/Nanjing/6/2013_KF007117
A/Zhejiang/HZ1/2013_KF055465
94
A/Jiangsu/2/2013_KF226108
87
A/chicken/Jiangsu/SC099/2013_CY146933
A/Jiangsu/1/2013_KF278749
A/Wuxi/4/2013_KF007077
A/Zhenjiang/1/2013_KF007061
A/chicken/Zhejiang/C483/2013_KF042084
A/Shanghai/JS01/2013_KF609512
92
A/chicken/Shanghai/S1358/2013_CY147013
A/chicken/Zhejiang/SD007/2013_CY147037
A/Jiangsu/1/2013_KF226116
71
A/Nanjing/1/2013_KC896775
A/chicken/Shanghai/S1079/2013_CY146997
99
A/Changsha/1/2013_KF420302
A/Nanchang/1/2013_KF277193
A/chicken/Jiangxi/SD001/2013_CY146949
82
A/pigeon/Shanghai/S1069/2013_CY147173
A/chicken/Shanghai/S1078/2013_CY146989
A/chicken/Shanghai/S1076/2013_CY146973
A/chicken/Shanghai/019/2013_KF542883
A/chicken/Shanghai/S1077/2013_CY146981
A/Changsha/2/2013_KF420303
A/Hong Kong/470129/2013_KF952509
100
A/pigeon/Zhejiang/P1/2013_KF042081
A/pigeon/Zhejiang/P2/2013_KF042082
A/chicken/Shanghai/S1080/2013_CY147005
100
A/Huizhou/01/2013_KF667740
A/Guangdong/1/2013_KF662948
74
A/Guangdong/03/2013_KJ023068
A/Guangdong/05/2013_KJ023083
A/Guangdong/02/2013_KF944961
A/Guangdong/04/2013_KJ023075
0.005

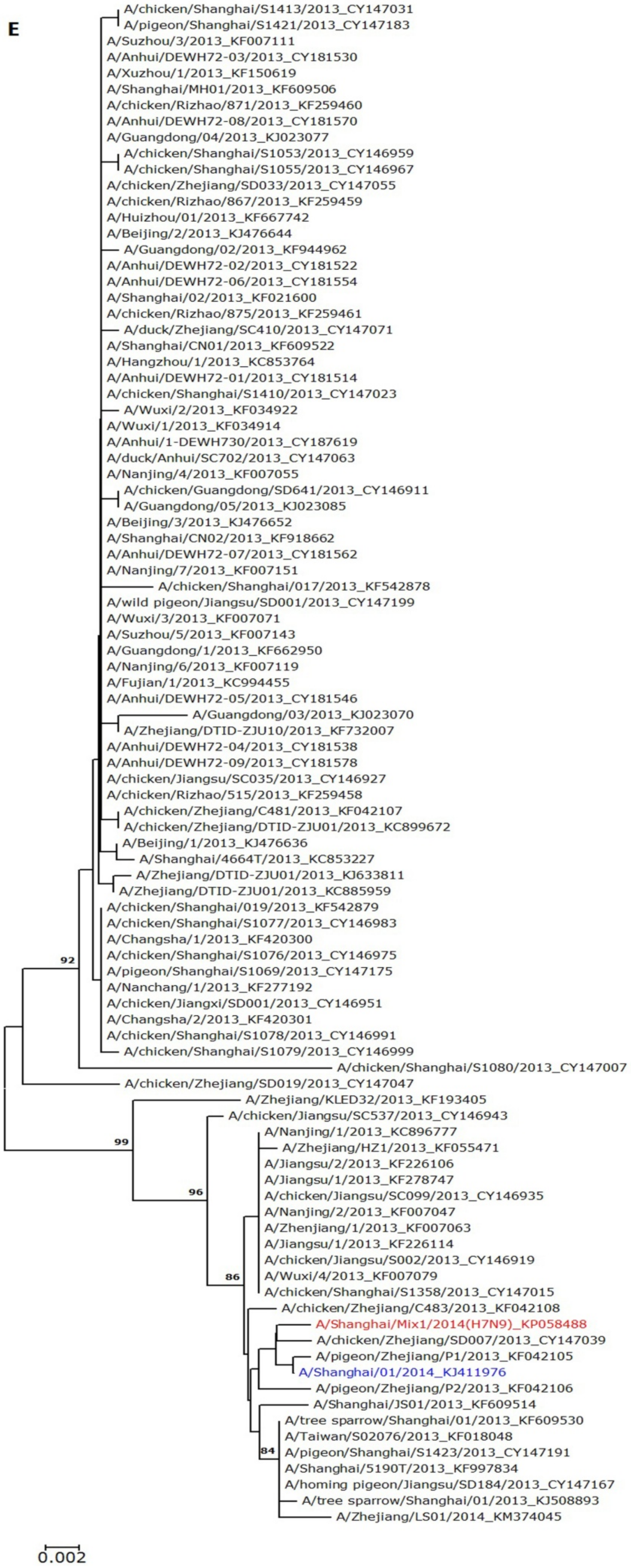
E
A/chicken/Shanghai/S1413/2013_CY147031
A/pigeon/Shanghai/S1421/2013_CY147183
A/Suzhou/3/2013_KF007111
A/Anhui/DEWH72-03/2013_CY181530
A/Xuzhou/1/2013_KF150619
A/Shanghai/MH01/2013_KF609506
A/chicken/Rizhao/871/2013_KF259460
A/Anhui/DEWH72-08/2013_CY181570
A/Guangdong/04/2013_KJ023077
A/chicken/Shanghai/S1053/2013_CY146959
A/chicken/Shanghai/S1055/2013_CY146967
A/chicken/Zhejiang/SD033/2013_CY147055
A/chicken/Rizhao/867/2013_KF259459
A/Huizhou/01/2013_KF667742
A/Beijing/2/2013_KJ476644
A/Guangdong/02/2013_KF944962
A/Anhui/DEWH72-02/2013_CY181522
A/Anhui/DEWH72-06/2013_CY181554
A/Shanghai/02/2013_KF021600
A/chicken/Rizhao/875/2013_KF259461
A/duck/Zhejiang/SC410/2013_CY147071
A/Shanghai/CN01/2013_KF609522
A/Hangzhou/1/2013_KC853764
A/Anhui/DEWH72-01/2013_CY181514
A/chicken/Shanghai/S1410/2013_CY147023
A/Wuxi/2/2013_KF034922
A/Wuxi/1/2013_KF034914
A/Anhui/1-DEWH730/2013_CY187619
A/duck/Anhui/SC702/2013_CY147063
A/Nanjing/4/2013_KF007055
A/chicken/Guangdong/SD641/2013_CY146911
A/Guangdong/05/2013_KJ023085
A/Beijing/3/2013_KJ476652
A/Shanghai/CN02/2013_KF918662
A/Anhui/DEWH72-07/2013_CY181562
A/Nanjing/7/2013_KF007151
A/chicken/Shanghai/017/2013_KF542878
A/wild pigeon/Jiangsu/SD001/2013_CY147199
A/Wuxi/3/2013_KF007071
A/Suzhou/5/2013_KF007143
A/Guangdong/1/2013_KF662950
A/Nanjing/6/2013_KF007119
A/Fujian/1/2013_KC994455
A/Anhui/DEWH72-05/2013_CY181546
A/Guangdong/03/2013_KJ023070
A/Zhejiang/DTID-ZJU10/2013_KF732007
A/Anhui/DEWH72-04/2013_CY181538
A/Anhui/DEWH72-09/2013_CY181578
A/chicken/Jiangsu/SC035/2013_CY146927
A/chicken/Rizhao/515/2013_KF259458
A/chicken/Zhejiang/C481/2013_KF042107
A/chicken/Zhejiang/DTID-ZJU01/2013_KC899672
A/Beijing/1/2013_KJ476636
A/Shanghai/4664T/2013_KC853227
A/Zhejiang/DTID-ZJU01/2013_KJ633811
A/Zhejiang/DTID-ZJU01/2013_KC885959
A/chicken/Shanghai/019/2013_KF542879
A/chicken/Shanghai/S1077/2013_CY146983
A/Changsha/1/2013_KF420300
92
A/chicken/Shanghai/S1076/2013_CY146975
A/pigeon/Shanghai/S1069/2013_CY147175
A/Nanchang/1/2013_KF277192
A/chicken/Jiangxi/SD001/2013_CY146951
A/Changsha/2/2013_KF420301
A/chicken/Shanghai/S1078/2013_CY146991
A/chicken/Shanghai/S1079/2013_CY146999
A/chicken/Shanghai/S1080/2013_CY147007
A/chicken/Zhejiang/SD019/2013_CY147047
A/Zhejiang/KLED32/2013_KF193405
A/chicken/Jiangsu/SC537/2013_CY146943
A/Nanjing/1/2013_KC896777
99
A/Zhejiang/HZ1/2013_KF055471
A/Jiangsu/2/2013_KF226106
A/Jiangsu/1/2013_KF278747
96
A/chicken/Jiangsu/SC099/2013_CY146935
A/Nanjing/2/2013_KF007047
A/Zhenjiang/1/2013_KF007063
A/Jiangsu/1/2013_KF226114
A/chicken/Jiangsu/S002/2013_CY146919
86
A/Wuxi/4/2013_KF007079
A/chicken/Shanghai/S1358/2013_CY147015
A/chicken/Zhejiang/C483/2013_KF042108
A/Shanghai/Mix1/2014(H7N9)_KP058488
A/chicken/Zhejiang/SD007/2013_CY147039
A/pigeon/Zhejiang/P1/2013_KF042105
A/Shanghai/01/2014_KJ411976
A/pigeon/Zhejiang/P2/2013_KF042106
A/Shanghai/JS01/2013_KF609514
A/tree sparrow/Shanghai/01/2013_KF609530
A/Taiwan/S02076/2013_KF018048
84
A/pigeon/Shanghai/S1423/2013_CY147191
A/Shanghai/5190T/2013_KF997834
A/homing pigeon/Jiangsu/SD184/2013_CY147167
A/tree sparrow/Shanghai/01/2013_KJ508893
A/Zhejiang/LS01/2014_KM374045
0.002

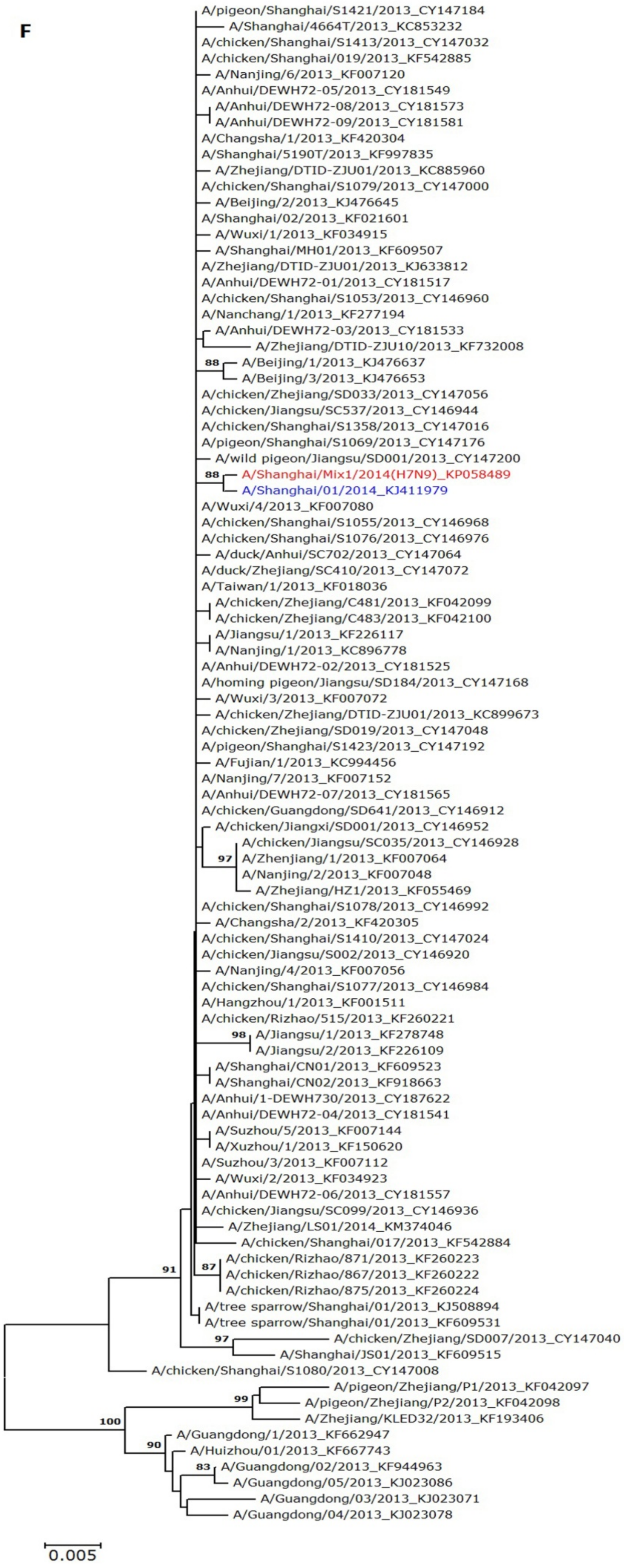
F
A/pigeon/Shanghai/S1421/2013_CY147184
A/Shanghai/4664T/2013_KC853232
A/chicken/Shanghai/S1413/2013_CY147032
A/chicken/Shanghai/019/2013_KF542885
A/Nanjing/6/2013_KF007120
A/Anhui/DEWH72-05/2013_CY181549
A/Anhui/DEWH72-08/2013_CY181573
A/Anhui/DEWH72-09/2013_CY181581
A/Changsha/1/2013_KF420304
A/Shanghai/5190T/2013_KF997835
A/Zhejiang/DTID-ZJU01/2013_KC885960
A/chicken/Shanghai/S1079/2013_CY147000
A/Beijing/2/2013_KJ476645
A/Shanghai/02/2013_KF021601
A/Wuxi/1/2013_KF034915
A/Shanghai/MH01/2013_KF609507
A/Zhejiang/DTID-ZJU01/2013_KJ633812
A/Anhui/DEWH72-01/2013_CY181517
A/chicken/Shanghai/S1053/2013_CY146960
A/Nanchang/1/2013_KF277194
A/Anhui/DEWH72-03/2013_CY181533
A/Zhejiang/DTID-ZJU10/2013_KF732008
88
A/Beijing/1/2013_KJ476637
A/Beijing/3/2013_KJ476653
A/chicken/Zhejiang/SD033/2013_CY147056
A/chicken/Jiangsu/SC537/2013_CY146944
A/chicken/Shanghai/S1358/2013_CY147016
A/pigeon/Shanghai/S1069/2013_CY147176
A/wild pigeon/Jiangsu/SD001/2013_CY147200
88
A/Shanghai/Mix1/2014(H7N9)_KP058489
A/Shanghai/01/2014_KJ411979
A/Wuxi/4/2013_KF007080
A/chicken/Shanghai/S1055/2013_CY146968
A/chicken/Shanghai/S1076/2013_CY146976
A/duck/Anhui/SC702/2013_CY147064
A/duck/Zhejiang/SC410/2013_CY147072
A/Taiwan/1/2013_KF018036
A/chicken/Zhejiang/C481/2013_KF042099
A/chicken/Zhejiang/C483/2013_KF042100
A/Jiangsu/1/2013_KF226117
A/Nanjing/1/2013_KC896778
A/Anhui/DEWH72-02/2013_CY181525
A/homing pigeon/Jiangsu/SD184/2013_CY147168
A/Wuxi/3/2013_KF007072
A/chicken/Zhejiang/DTID-ZJU01/2013_KC899673
A/chicken/Zhejiang/SD019/2013_CY147048
A/pigeon/Shanghai/S1423/2013_CY147192
A/Fujian/1/2013_KC994456
A/Nanjing/7/2013_KF007152
A/Anhui/DEWH72-07/2013_CY181565
A/chicken/Guangdong/SD641/2013_CY146912
A/chicken/Jiangxi/SD001/2013_CY146952
A/chicken/Jiangsu/SC035/2013_CY146928
97
A/Zhenjiang/1/2013_KF007064
A/Nanjing/2/2013_KF007048
A/Zhejiang/HZ1/2013_KF055469
A/chicken/Shanghai/S1078/2013_CY146992
A/Changsha/2/2013_KF420305
A/chicken/Shanghai/S1410/2013_CY147024
A/chicken/Jiangsu/S002/2013_CY146920
A/Nanjing/4/2013_KF007056
A/chicken/Shanghai/S1077/2013_CY146984
A/Hangzhou/1/2013_KF001511
A/chicken/Rizhao/515/2013_KF260221
98
A/Jiangsu/1/2013_KF278748
A/Jiangsu/2/2013_KF226109
A/Shanghai/CN01/2013_KF609523
A/Shanghai/CN02/2013_KF918663
A/Anhui/1-DEWH730/2013_CY187622
A/Anhui/DEWH72-04/2013_CY181541
A/Suzhou/5/2013_KF007144
A/Xuzhou/1/2013_KF150620
A/Suzhou/3/2013_KF007112
A/Wuxi/2/2013_KF034923
A/Anhui/DEWH72-06/2013_CY181557
A/chicken/Jiangsu/SC099/2013_CY146936
A/Zhejiang/LS01/2014_KM374046
A/chicken/Shanghai/017/2013_KF542884
87
A/chicken/Rizhao/871/2013_KF260223
A/chicken/Rizhao/867/2013_KF260222
A/chicken/Rizhao/875/2013_KF260224
91
A/tree sparrow/Shanghai/01/2013_KJ508894
A/tree sparrow/Shanghai/01/2013_KF609531
97
A/chicken/Zhejiang/SD007/2013_CY147040
A/Shanghai/JS01/2013_KF609515
A/chicken/Shanghai/S1080/2013_CY147008
99
A/pigeon/Zhejiang/P1/2013_KF042097
A/pigeon/Zhejiang/P2/2013_KF042098
100
A/Zhejiang/KLED32/2013_KF193406
90
A/Guangdong/1/2013_KF662947
A/Huizhou/01/2013_KF667743
83
A/Guangdong/02/2013_KF944963
A/Guangdong/05/2013_KJ023086
A/Guangdong/03/2013_KJ023071
A/Guangdong/04/2013_KJ023078
0.005

G

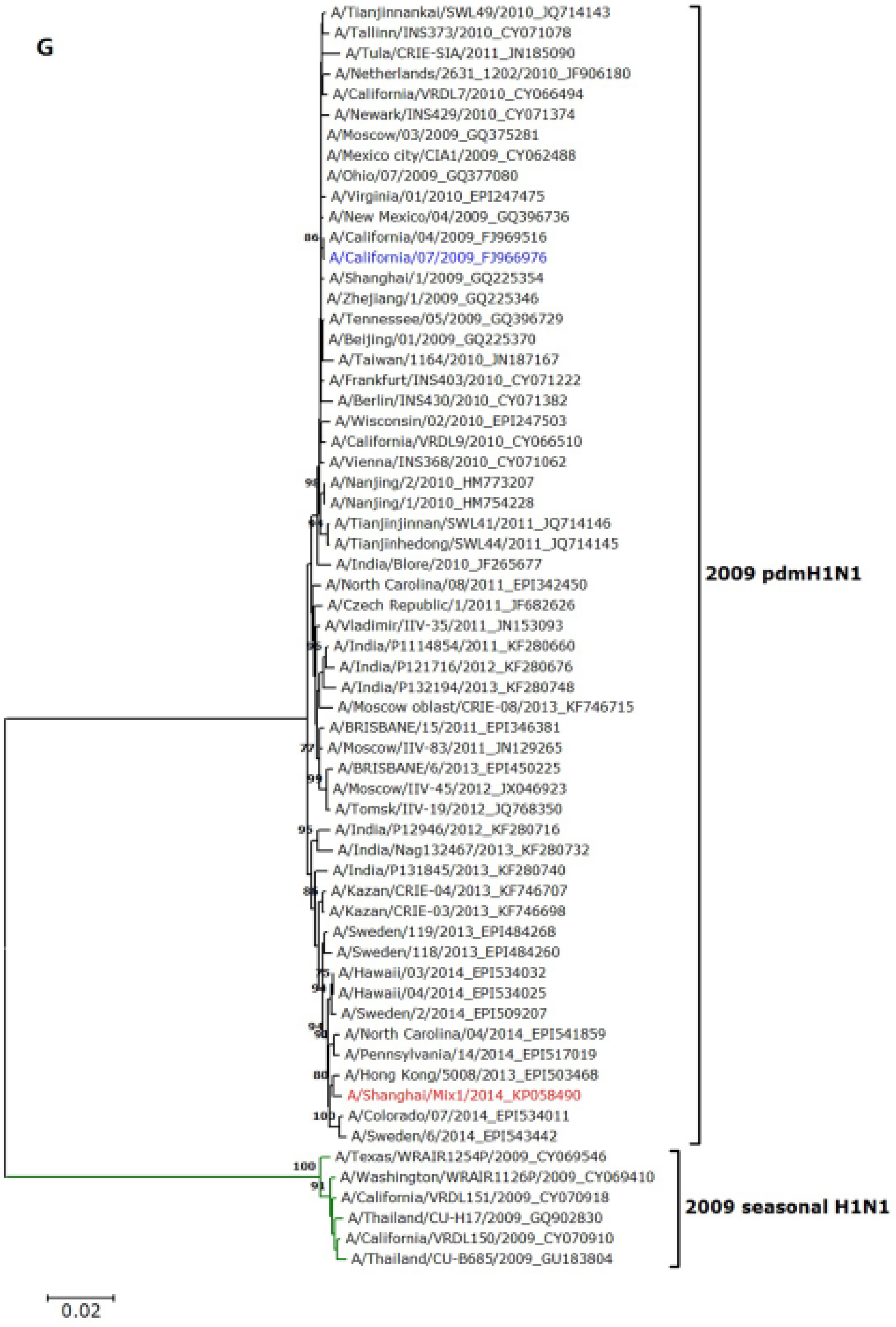
A/Tianjinnankai/SWL49/2010_JQ714143
A/Tallinn/INS373/2010_CY071078
A/Tula/CRIE-SIA/2011_JN185090
A/Netherlands/2631_1202/2010_JF906180
A/California/VRDL7/2010_CY066494
A/Newark/INS429/2010_CY071374
A/Moscow/03/2009_GQ375281
A/Mexico city/CIA1/2009_CY062488
A/Ohio/07/2009_GQ377080
A/Virginia/01/2010_EPI247475
A/New Mexico/04/2009_GQ396736
A/California/04/2009_FJ969516
A/California/07/2009_FJ966976
A/Shanghai/1/2009_GQ225354
A/Zhejiang/1/2009_GQ225346
A/Tennessee/05/2009_GQ396729
A/Beijing/01/2009_GQ225370
A/Taiwan/1164/2010_JN187167
A/Frankfurt/INS403/2010_CY071222
A/Berlin/INS430/2010_CY071382
A/Wisconsin/02/2010_EPI247503
A/California/VRDL9/2010_CY066510
A/Vienna/INS368/2010_CY071062
A/Nanjing/2/2010_HM773207
A/Nanjing/1/2010_HM754228
A/Tianjinjinnan/SWL41/2011_JQ714146
A/Tianjinhedong/SWL44/2011_JQ714145
A/India/Blore/2010_JF265677
A/North Carolina/08/2011_EPI342450
A/Czech Republic/1/2011_JF682626
A/Vladimir/IIV-35/2011_JN153093
A/India/P1114854/2011_KF280660
A/India/P121716/2012_KF280676
A/India/P132194/2013_KF280748
A/Moscow oblast/CRIE-08/2013_KF746715
A/BRISBANE/15/2011_EPI346381
A/Moscow/IIV-83/2011_JN129265
A/BRISBANE/6/2013_EPI450225
A/Moscow/IIV-45/2012_JX046923
A/Tomsk/IIV-19/2012_JQ768350
A/India/P12946/2012_KF280716
A/India/Nag132467/2013_KF280732
A/India/P131845/2013_KF280740
A/Kazan/CRIE-04/2013_KF746707
A/Kazan/CRIE-03/2013_KF746698
A/Sweden/119/2013_EPI484268
A/Sweden/118/2013_EPI484260
A/Hawaii/03/2014_EPI534032
A/Hawaii/04/2014_EPI534025
A/Sweden/2/2014_EPI509207
A/North Carolina/04/2014_EPI541859
A/Pennsylvania/14/2014_EPI517019
A/Hong Kong/5008/2013_EPI503468
A/Shanghai/Mix1/2014_KP058490
A/Colorado/07/2014_EPI534011
A/Sweden/6/2014_EPI543442
2009 pdmH1N1
A/Texas/WRAIR1254P/2009_CY069546
A/Washington/WRAIR1126P/2009_CY069410
A/California/VRDL151/2009_CY070918
A/Thailand/CU-H17/2009_GQ902830
A/California/VRDL150/2009_CY070910
A/Thailand/CU-B685/2009_GU183804
2009 seasonal H1N1
0.02

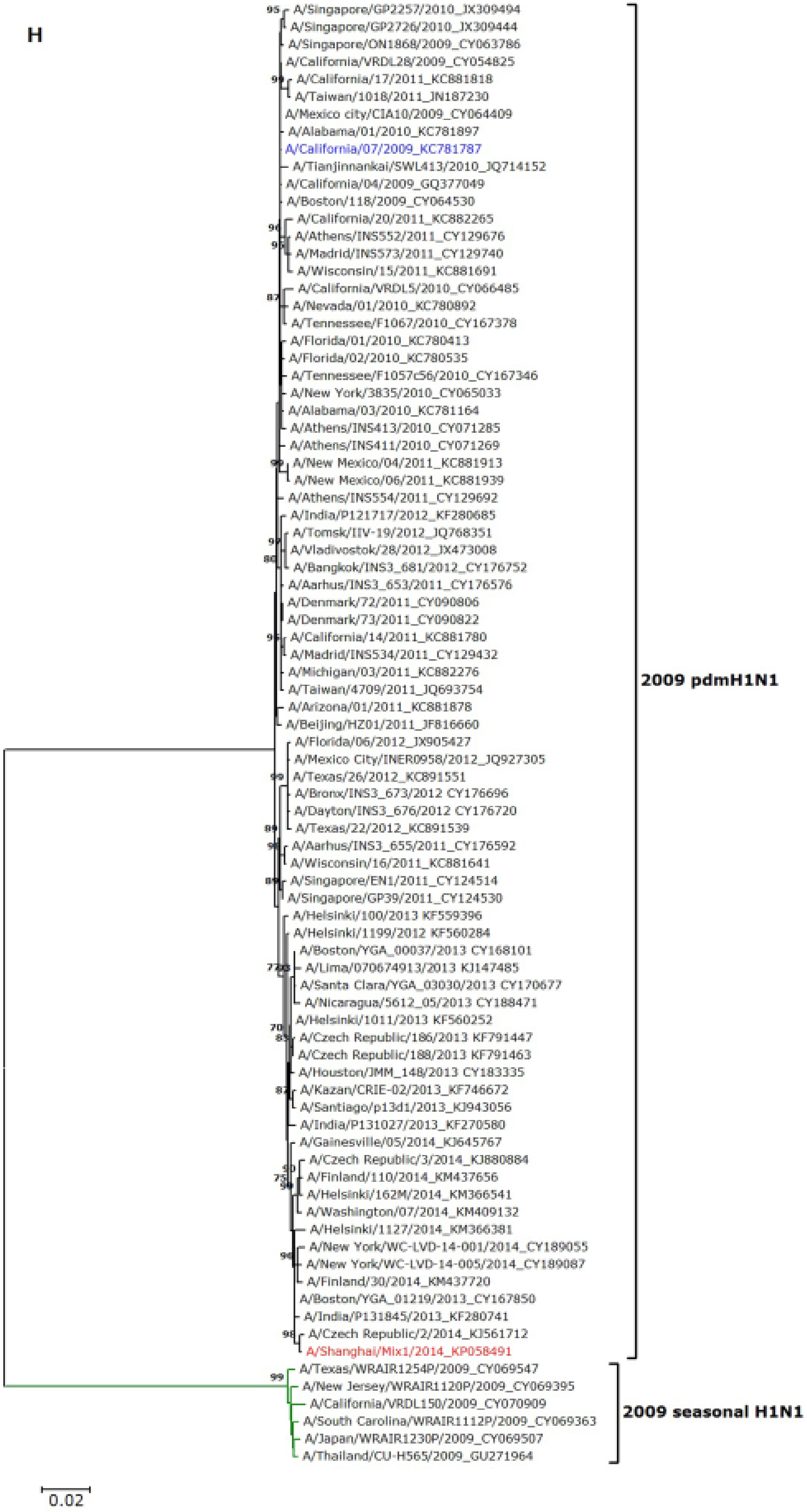
H
A/Singapore/GP2257/2010_JX309494
A/Singapore/GP2726/2010_JX309444
A/Singapore/ON1868/2009_CY063786
A/California/VRDL28/2009_CY054825
A/California/17/2011_KC881818
A/Taiwan/1018/2011_JN187230
A/Mexico city/CIA10/2009_CY064409
A/Alabama/01/2010_KC781897
A/California/07/2009_KC781787
A/Tianjinnankai/SWL413/2010_JQ714152
A/California/04/2009_GQ377049
A/Boston/118/2009_CY064530
A/California/20/2011_KC882265
A/Athens/INS552/2011_CY129676
A/Madrid/INS573/2011_CY129740
A/Wisconsin/15/2011_KC881691
A/California/VRDL5/2010_CY066485
A/Nevada/01/2010_KC780892
A/Tennessee/F1067/2010_CY167378
A/Florida/01/2010_KC780413
A/Florida/02/2010_KC780535
A/Tennessee/F1057c56/2010_CY167346
A/New York/3835/2010_CY065033
A/Alabama/03/2010_KC781164
A/Athens/INS413/2010_CY071285
A/Athens/INS411/2010_CY071269
A/New Mexico/04/2011_KC881913
A/New Mexico/06/2011_KC881939
A/Athens/INS554/2011_CY129692
A/India/P121717/2012_KF280685
A/Tomsk/IIV-19/2012_JQ768351
A/Vladivostok/28/2012_JX473008
A/Bangkok/INS3_681/2012_CY176752
A/Aarhus/INS3_653/2011_CY176576
A/Denmark/72/2011_CY090806
A/Denmark/73/2011_CY090822
A/California/14/2011_KC881780
A/Madrid/INS534/2011_CY129432
A/Michigan/03/2011_KC882276
A/Taiwan/4709/2011_JQ693754
A/Arizona/01/2011_KC881878
A/Beijing/HZ01/2011_JF816660
A/Florida/06/2012_JX905427
A/Mexico City/INER0958/2012_JQ927305
A/Texas/26/2012_KC891551
A/Bronx/INS3_673/2012 CY176696
A/Dayton/INS3_676/2012 CY176720
A/Texas/22/2012_KC891539
A/Aarhus/INS3_655/2011_CY176592
A/Wisconsin/16/2011_KC881641
A/Singapore/EN1/2011_CY124514
A/Singapore/GP39/2011_CY124530
A/Helsinki/100/2013 KF559396
A/Helsinki/1199/2012 KF560284
A/Boston/YGA_00037/2013 CY168101
A/Lima/070674913/2013 KJ147485
A/Santa Clara/YGA_03030/2013 CY170677
A/Nicaragua/5612_05/2013 CY188471
A/Helsinki/1011/2013 KF560252
A/Czech Republic/186/2013 KF791447
A/Czech Republic/188/2013 KF791463
A/Houston/JMM_148/2013 CY183335
A/Kazan/CRIE-02/2013_KF746672
A/Santiago/p13d1/2013_KJ943056
A/India/P131027/2013_KF270580
A/Gainesville/05/2014_KJ645767
A/Czech Republic/3/2014_KJ880884
A/Finland/110/2014_KM437656
A/Helsinki/162M/2014_KM366541
A/Washington/07/2014_KM409132
A/Helsinki/1127/2014_KM366381
A/New York/WC-LVD-14-001/2014_CY189055
A/New York/WC-LVD-14-005/2014_CY189087
A/Finland/30/2014_KM437720
A/Boston/YGA_01219/2013_CY167850
A/India/P131845/2013_KF280741
A/Czech Republic/2/2014_KJ561712
A/Shanghai/Mix1/2014_KP058491
2009 pdmH1N1
A/Texas/WRAIR1254P/2009_CY069547
A/New Jersey/WRAIR1120P/2009_CY069395
A/California/VRDL150/2009_CY070909
A/South Carolina/WRAIR1112P/2009_CY069363
A/Japan/WRAIR1230P/2009_CY069507
A/Thailand/CU-H565/2009_GU271964
2009 seasonal H1N1
0.02

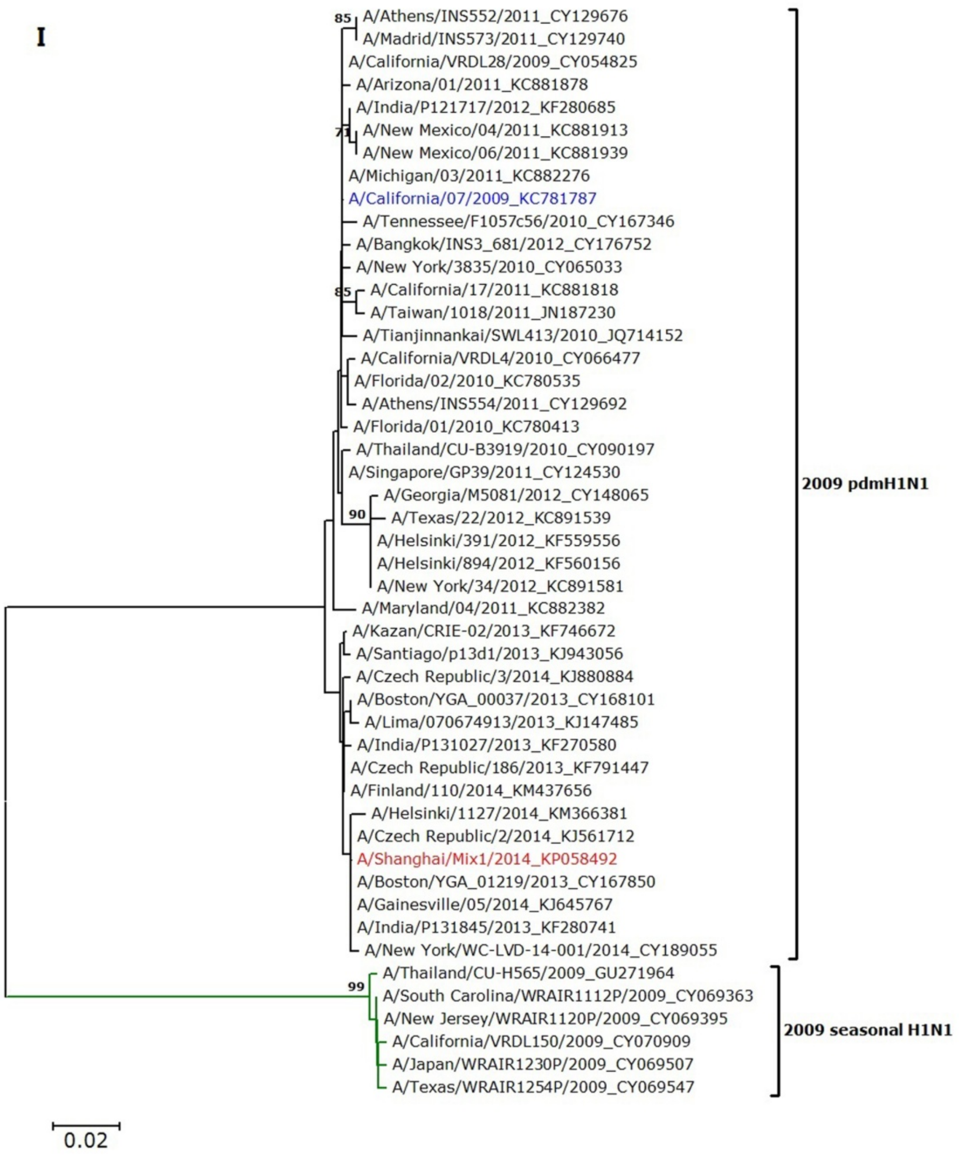
I
85
A/Athens/INS552/2011_CY129676
A/Madrid/INS573/2011_CY129740
A/California/VRDL28/2009_CY054825
A/Arizona/01/2011_KC881878
A/India/P121717/2012_KF280685
71
A/New Mexico/04/2011_KC881913
A/New Mexico/06/2011_KC881939
A/Michigan/03/2011_KC882276
A/California/07/2009_KC781787
A/Tennessee/F1057c56/2010_CY167346
A/Bangkok/INS3_681/2012_CY176752
A/New York/3835/2010_CY065033
85
A/California/17/2011_KC881818
A/Taiwan/1018/2011_JN187230
A/Tianjinnankai/SWL413/2010_JQ714152
A/California/VRDL4/2010_CY066477
A/Florida/02/2010_KC780535
A/Athens/INS554/2011_CY129692
A/Florida/01/2010_KC780413
A/Thailand/CU-B3919/2010_CY090197
A/Singapore/GP39/2011_CY124530
A/Georgia/M5081/2012_CY148065
90
A/Texas/22/2012_KC891539
A/Helsinki/391/2012_KF559556
A/Helsinki/894/2012_KF560156
A/New York/34/2012_KC891581
A/Maryland/04/2011_KC882382
A/Kazan/CRIE-02/2013_KF746672
A/Santiago/p13d1/2013_KJ943056
A/Czech Republic/3/2014_KJ880884
A/Boston/YGA_00037/2013_CY168101
A/Lima/070674913/2013_KJ147485
A/India/P131027/2013_KF270580
A/Czech Republic/186/2013_KF791447
A/Finland/110/2014_KM437656
A/Helsinki/1127/2014_KM366381
A/Czech Republic/2/2014_KJ561712
A/Shanghai/Mix1/2014_KP058492
A/Boston/YGA_01219/2013_CY167850
A/Gainesville/05/2014_KJ645767
A/India/P131845/2013_KF280741
A/New York/WC-LVD-14-001/2014_CY189055
2009 pdmH1N1
99
A/Thailand/CU-H565/2009_GU271964
A/South Carolina/WRAIR1112P/2009_CY069363
A/New Jersey/WRAIR1120P/2009_CY069395
A/California/VRDL150/2009_CY070909
A/Japan/WRAIR1230P/2009_CY069507
A/Texas/WRAIR1254P/2009_CY069547
2009 seasonal H1N1
0.02

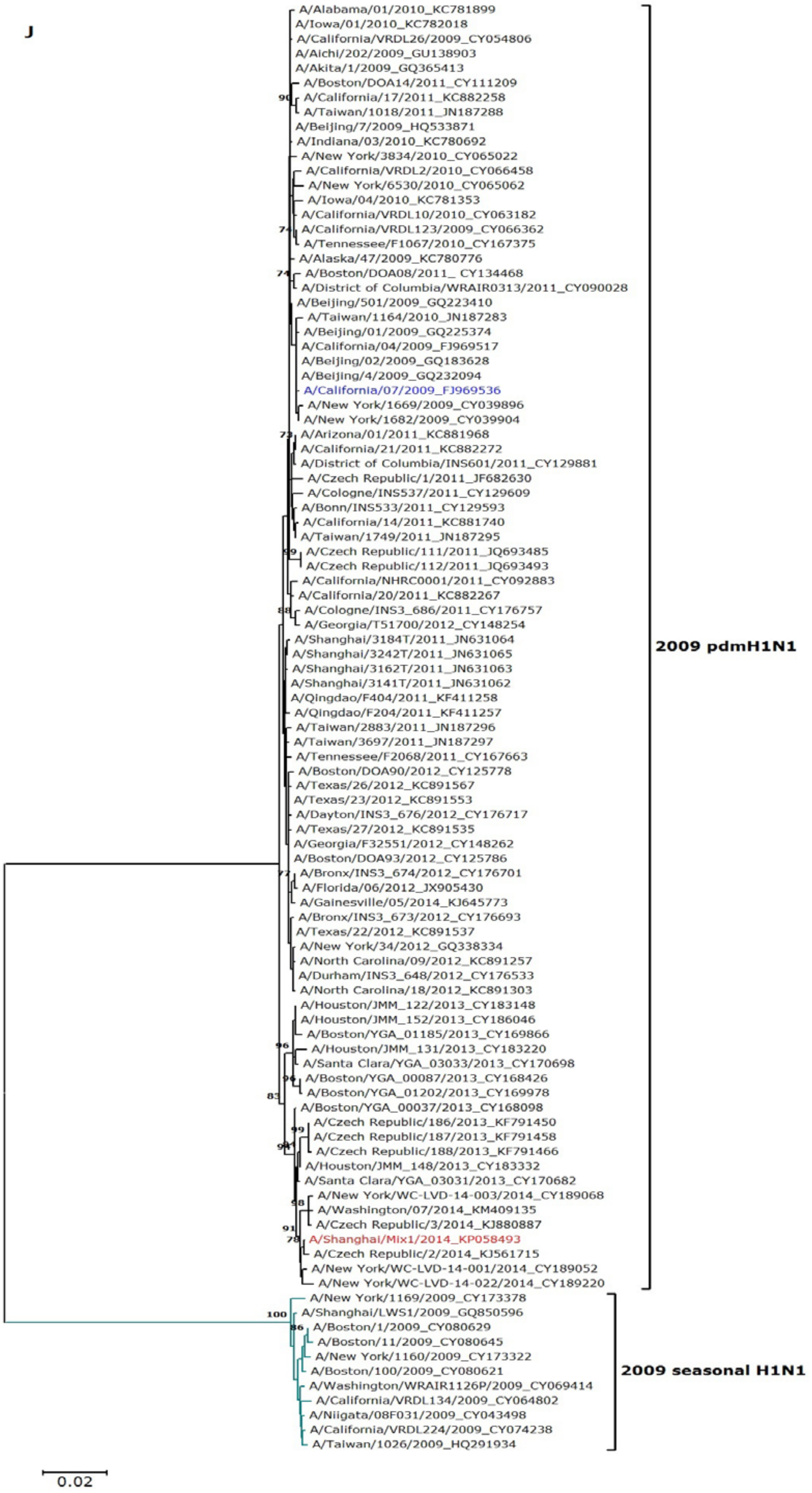
J
A/Alabama/01/2010_KC781899
A/Iowa/01/2010_KC782018
A/California/VRDL26/2009_CY054806
A/Aichi/202/2009_GU138903
A/Akita/1/2009_GQ365413
A/Boston/DOA14/2011_CY111209
A/California/17/2011_KC882258
A/Taiwan/1018/2011_JN187288
A/Beijing/7/2009_HQ533871
A/Indiana/03/2010_KC780692
A/New York/3834/2010_CY065022
A/California/VRDL2/2010_CY066458
A/New York/6530/2010_CY065062
A/Iowa/04/2010_KC781353
A/California/VRDL10/2010_CY063182
A/California/VRDL123/2009_CY066362
A/Tennessee/F1067/2010_CY167375
A/Alaska/47/2009_KC780776
A/Boston/DOA08/2011_ CY134468
A/District of Columbia/WRAIR0313/2011_CY090028
A/Beijing/501/2009_GQ223410
A/Taiwan/1164/2010_JN187283
A/Beijing/01/2009_GQ225374
A/California/04/2009_FJ969517
A/Beijing/02/2009_GQ183628
A/Beijing/4/2009_GQ232094
A/California/07/2009_FJ969536
A/New York/1669/2009_CY039896
A/New York/1682/2009_CY039904
A/Arizona/01/2011_KC881968
A/California/21/2011_KC882272
A/District of Columbia/INS601/2011_CY129881
A/Czech Republic/1/2011_JF682630
A/Cologne/INS537/2011_CY129609
A/Bonn/INS533/2011_CY129593
A/California/14/2011_KC881740
A/Taiwan/1749/2011_JN187295
A/Czech Republic/111/2011_JQ693485
A/Czech Republic/112/2011_JQ693493
A/California/NHRC0001/2011_CY092883
A/California/20/2011_KC882267
A/Cologne/INS3_686/2011_CY176757
A/Georgia/T51700/2012_CY148254
A/Shanghai/3184T/2011_JN631064
A/Shanghai/3242T/2011_JN631065
A/Shanghai/3162T/2011_JN631063
A/Shanghai/3141T/2011_JN631062
A/Qingdao/F404/2011_KF411258
A/Qingdao/F204/2011_KF411257
A/Taiwan/2883/2011_JN187296
A/Taiwan/3697/2011_JN187297
A/Tennessee/F2068/2011_CY167663
A/Boston/DOA90/2012_CY125778
A/Texas/26/2012_KC891567
A/Texas/23/2012_KC891553
A/Dayton/INS3_676/2012_CY176717
A/Texas/27/2012_KC891535
A/Georgia/F32551/2012_CY148262
A/Boston/DOA93/2012_CY125786
A/Bronx/INS3_674/2012_CY176701
A/Florida/06/2012_JX905430
A/Gainesville/05/2014_KJ645773
A/Bronx/INS3_673/2012_CY176693
A/Texas/22/2012_KC891537
A/New York/34/2012_GQ338334
A/North Carolina/09/2012_KC891257
A/Durham/INS3_648/2012_CY176533
A/North Carolina/18/2012_KC891303
A/Houston/JMM_122/2013_CY183148
A/Houston/JMM_152/2013_CY186046
A/Boston/YGA_01185/2013_CY169866
A/Houston/JMM_131/2013_CY183220
A/Santa Clara/YGA_03033/2013_CY170698
A/Boston/YGA_00087/2013_CY168426
A/Boston/YGA_01202/2013_CY169978
A/Boston/YGA_00037/2013_CY168098
A/Czech Republic/186/2013_KF791450
A/Czech Republic/187/2013_KF791458
A/Czech Republic/188/2013_KF791466
A/Houston/JMM_148/2013_CY183332
A/Santa Clara/YGA_03031/2013_CY170682
A/New York/WC-LVD-14-003/2014_CY189068
A/Washington/07/2014_KM409135
A/Czech Republic/3/2014_KJ880887
A/Shanghai/Mix1/2014_KP058493
A/Czech Republic/2/2014_KJ561715
A/New York/WC-LVD-14-001/2014_CY189052
A/New York/WC-LVD-14-022/2014_CY189220
2009 pdmH1N1
A/New York/1169/2009_CY173378
A/Shanghai/LWS1/2009_GQ850596
A/Boston/1/2009_CY080629
A/Boston/11/2009_CY080645
A/New York/1160/2009_CY173322
A/Boston/100/2009_CY080621
A/Washington/WRAIR1126P/2009_CY069414
A/California/VRDL134/2009_CY064802
A/Niigata/08F031/2009_CY043498
A/California/VRDL224/2009_CY074238
A/Taiwan/1026/2009_HQ291934
2009 seasonal H1N1
100
0.02

K

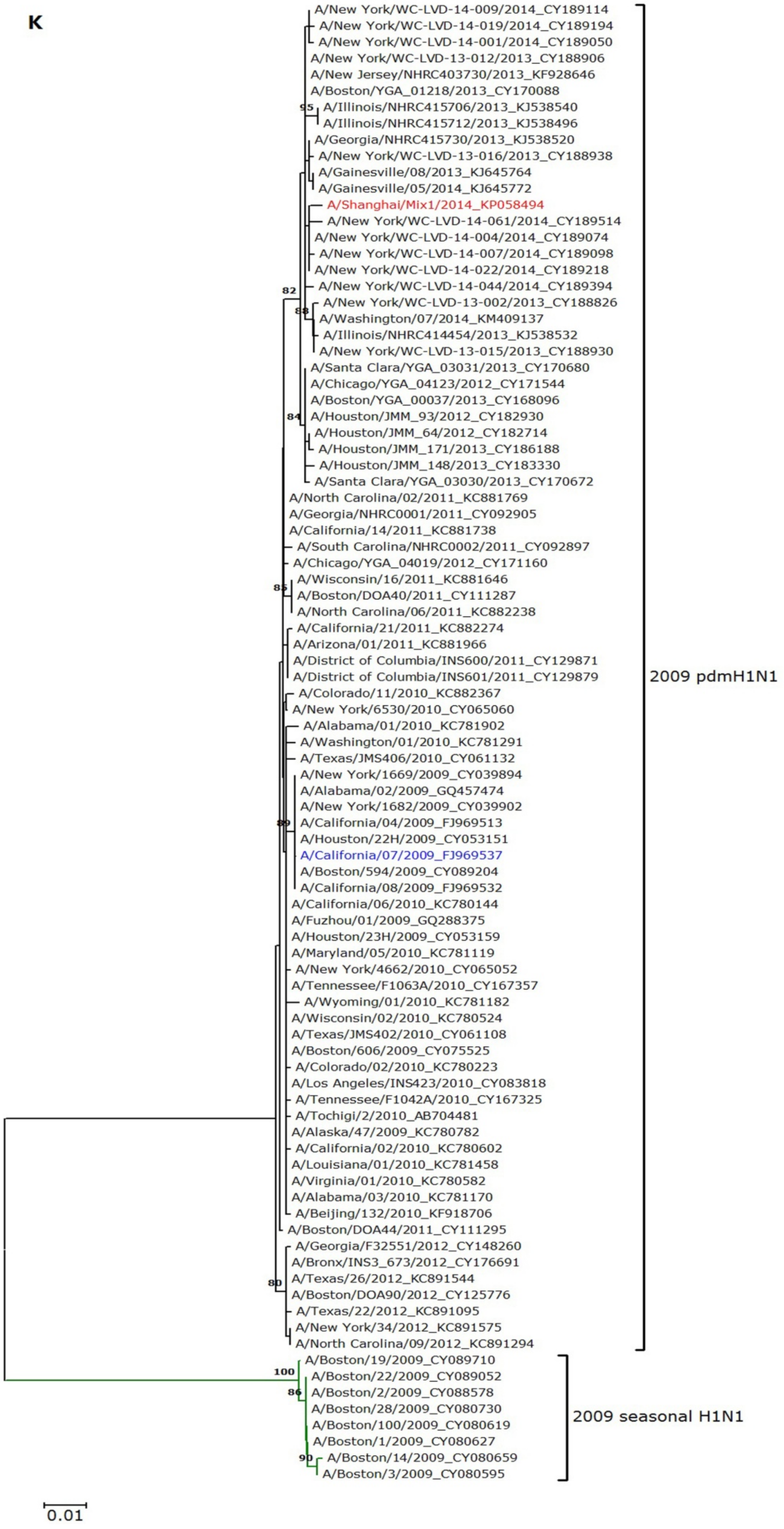
A/New York/WC-LVD-14-009/2014_CY189114
A/New York/WC-LVD-14-019/2014_CY189194
A/New York/WC-LVD-14-001/2014_CY189050
A/New York/WC-LVD-13-012/2013_CY188906
A/New Jersey/NHRC403730/2013_KF928646
A/Boston/YGA_01218/2013_CY170088
95
A/Illinois/NHRC415706/2013_KJ538540
A/Illinois/NHRC415712/2013_KJ538496
A/Georgia/NHRC415730/2013_KJ538520
A/New York/WC-LVD-13-016/2013_CY188938
A/Gainesville/08/2013_KJ645764
A/Gainesville/05/2014_KJ645772
A/Shanghai/Mix1/2014_KP058494
A/New York/WC-LVD-14-061/2014_CY189514
A/New York/WC-LVD-14-004/2014_CY189074
A/New York/WC-LVD-14-007/2014_CY189098
A/New York/WC-LVD-14-022/2014_CY189218
82
A/New York/WC-LVD-14-044/2014_CY189394
88
A/New York/WC-LVD-13-002/2013_CY188826
A/Washington/07/2014_KM409137
A/Illinois/NHRC414454/2013_KJ538532
A/New York/WC-LVD-13-015/2013_CY188930
A/Santa Clara/YGA_03031/2013_CY170680
A/Chicago/YGA_04123/2012_CY171544
A/Boston/YGA_00037/2013_CY168096
84
A/Houston/JMM_93/2012_CY182930
A/Houston/JMM_64/2012_CY182714
A/Houston/JMM_171/2013_CY186188
A/Houston/JMM_148/2013_CY183330
A/Santa Clara/YGA_03030/2013_CY170672
A/North Carolina/02/2011_KC881769
A/Georgia/NHRC0001/2011_CY092905
A/California/14/2011_KC881738
A/South Carolina/NHRC0002/2011_CY092897
A/Chicago/YGA_04019/2012_CY171160
85
A/Wisconsin/16/2011_KC881646
A/Boston/DOA40/2011_CY111287
A/North Carolina/06/2011_KC882238
A/California/21/2011_KC882274
A/Arizona/01/2011_KC881966
A/District of Columbia/INS600/2011_CY129871
A/District of Columbia/INS601/2011_CY129879
A/Colorado/11/2010_KC882367
A/New York/6530/2010_CY065060
A/Alabama/01/2010_KC781902
A/Washington/01/2010_KC781291
A/Texas/JMS406/2010_CY061132
A/New York/1669/2009_CY039894
A/Alabama/02/2009_GQ457474
A/New York/1682/2009_CY039902
89
A/California/04/2009_FJ969513
A/Houston/22H/2009_CY053151
A/California/07/2009_FJ969537
A/Boston/594/2009_CY089204
A/California/08/2009_FJ969532
A/California/06/2010_KC780144
A/Fuzhou/01/2009_GQ288375
A/Houston/23H/2009_CY053159
A/Maryland/05/2010_KC781119
A/New York/4662/2010_CY065052
A/Tennessee/F1063A/2010_CY167357
A/Wyoming/01/2010_KC781182
A/Wisconsin/02/2010_KC780524
A/Texas/JMS402/2010_CY061108
A/Boston/606/2009_CY075525
A/Colorado/02/2010_KC780223
A/Los Angeles/INS423/2010_CY083818
A/Tennessee/F1042A/2010_CY167325
A/Tochigi/2/2010_AB704481
A/Alaska/47/2009_KC780782
A/California/02/2010_KC780602
A/Louisiana/01/2010_KC781458
A/Virginia/01/2010_KC780582
A/Alabama/03/2010_KC781170
A/Beijing/132/2010_KF918706
A/Boston/DOA44/2011_CY111295
A/Georgia/F32551/2012_CY148260
A/Bronx/INS3_673/2012_CY176691
80
A/Texas/26/2012_KC891544
A/Boston/DOA90/2012_CY125776
A/Texas/22/2012_KC891095
A/New York/34/2012_KC891575
A/North Carolina/09/2012_KC891294
2009 pdmH1N1
100
A/Boston/19/2009_CY089710
A/Boston/22/2009_CY089052
86
A/Boston/2/2009_CY088578
A/Boston/28/2009_CY080730
A/Boston/100/2009_CY080619
A/Boston/1/2009_CY080627
90
A/Boston/14/2009_CY080659
A/Boston/3/2009_CY080595
2009 seasonal H1N1
0.01

L

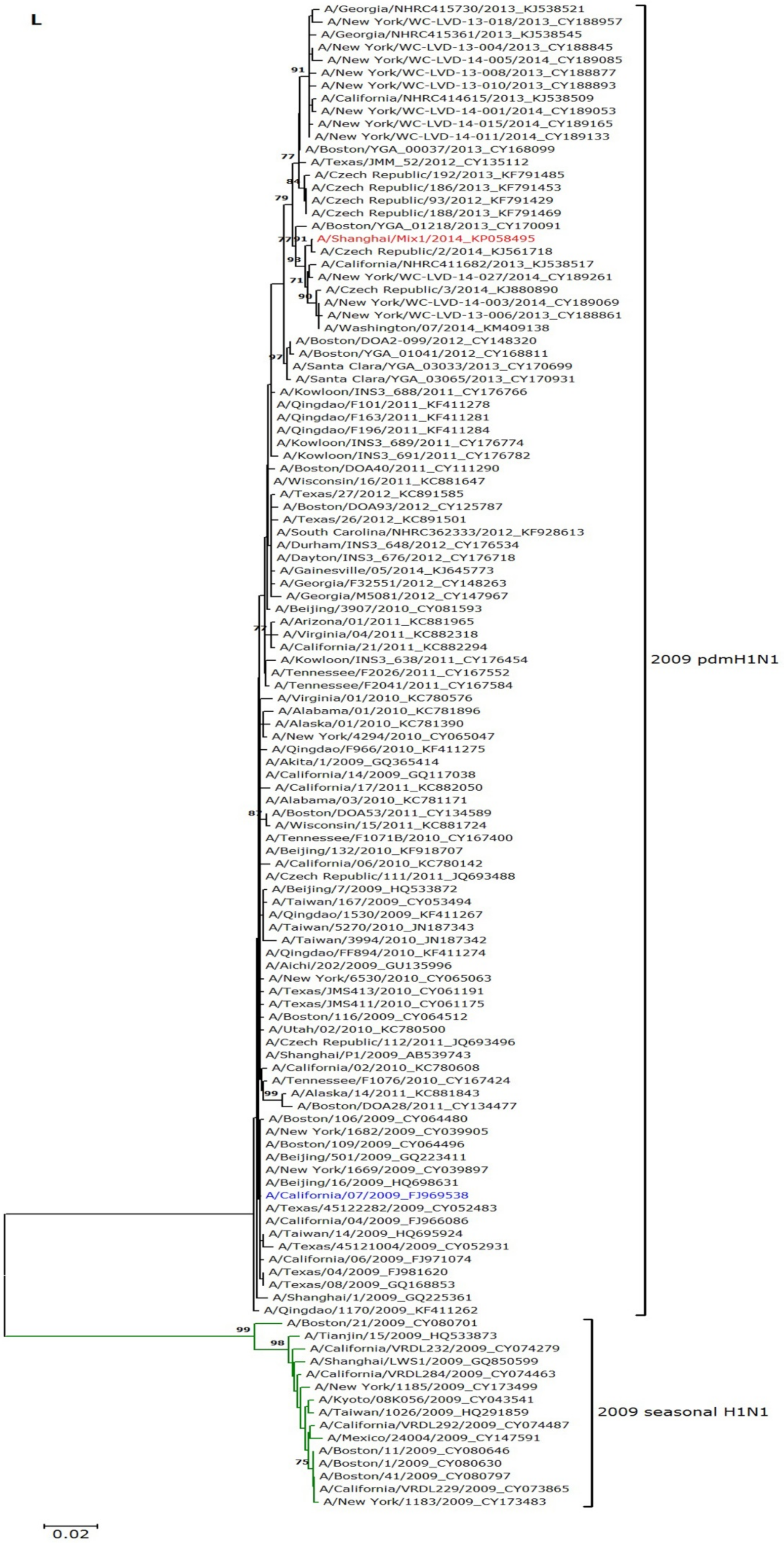
A/Georgia/NHRC415730/2013_KJ538521
A/New York/WC-LVD-13-018/2013_CY188957
A/Georgia/NHRC415361/2013_KJ538545
A/New York/WC-LVD-13-004/2013_CY188845
A/New York/WC-LVD-14-005/2014_CY189085
A/New York/WC-LVD-13-008/2013_CY188877
A/New York/WC-LVD-13-010/2013_CY188893
A/California/NHRC414615/2013_KJ538509
A/New York/WC-LVD-14-001/2014_CY189053
A/New York/WC-LVD-14-015/2014_CY189165
A/New York/WC-LVD-14-011/2014_CY189133
A/Boston/YGA_00037/2013_CY168099
A/Texas/JMM_52/2012_CY135112
A/Czech Republic/192/2013_KF791485
A/Czech Republic/186/2013_KF791453
A/Czech Republic/93/2012_KF791429
A/Czech Republic/188/2013_KF791469
A/Boston/YGA_01218/2013_CY170091
A/Shanghai/Mix1/2014_KP058495
A/Czech Republic/2/2014_KJ561718
A/California/NHRC411682/2013_KJ538517
A/New York/WC-LVD-14-027/2014_CY189261
A/Czech Republic/3/2014_KJ880890
A/New York/WC-LVD-14-003/2014_CY189069
A/New York/WC-LVD-13-006/2013_CY188861
A/Washington/07/2014_KM409138
A/Boston/DOA2-099/2012_CY148320
A/Boston/YGA_01041/2012_CY168811
A/Santa Clara/YGA_03033/2013_CY170699
A/Santa Clara/YGA_03065/2013_CY170931
A/Kowloon/INS3_688/2011_CY176766
A/Qingdao/F101/2011_KF411278
A/Qingdao/F163/2011_KF411281
A/Qingdao/F196/2011_KF411284
A/Kowloon/INS3_689/2011_CY176774
A/Kowloon/INS3_691/2011_CY176782
A/Boston/DOA40/2011_CY111290
A/Wisconsin/16/2011_KC881647
A/Texas/27/2012_KC891585
A/Boston/DOA93/2012_CY125787
A/Texas/26/2012_KC891501
A/South Carolina/NHRC362333/2012_KF928613
A/Durham/INS3_648/2012_CY176534
A/Dayton/INS3_676/2012_CY176718
A/Gainesville/05/2014_KJ645773
A/Georgia/F32551/2012_CY148263
A/Georgia/M5081/2012_CY147967
A/Beijing/3907/2010_CY081593
A/Arizona/01/2011_KC881965
A/Virginia/04/2011_KC882318
A/California/21/2011_KC882294
A/Kowloon/INS3_638/2011_CY176454
A/Tennessee/F2026/2011_CY167552
A/Tennessee/F2041/2011_CY167584
A/Virginia/01/2010_KC780576
A/Alabama/01/2010_KC781896
A/Alaska/01/2010_KC781390
A/New York/4294/2010_CY065047
A/Qingdao/F966/2010_KF411275
A/Akita/1/2009_GQ365414
A/California/14/2009_GQ117038
A/California/17/2011_KC882050
A/Alabama/03/2010_KC781171
A/Boston/DOA53/2011_CY134589
A/Wisconsin/15/2011_KC881724
A/Tennessee/F1071B/2010_CY167400
A/Beijing/132/2010_KF918707
A/California/06/2010_KC780142
A/Czech Republic/111/2011_JQ693488
A/Beijing/7/2009_HQ533872
A/Taiwan/167/2009_CY053494
A/Qingdao/1530/2009_KF411267
A/Taiwan/5270/2010_JN187343
A/Taiwan/3994/2010_JN187342
A/Qingdao/FF894/2010_KF411274
A/Aichi/202/2009_GU135996
A/New York/6530/2010_CY065063
A/Texas/JMS413/2010_CY061191
A/Texas/JMS411/2010_CY061175
A/Boston/116/2009_CY064512
A/Utah/02/2010_KC780500
A/Czech Republic/112/2011_JQ693496
A/Shanghai/P1/2009_AB539743
A/California/02/2010_KC780608
A/Tennessee/F1076/2010_CY167424
A/Alaska/14/2011_KC881843
A/Boston/DOA28/2011_CY134477
A/Boston/106/2009_CY064480
A/New York/1682/2009_CY039905
A/Boston/109/2009_CY064496
A/Beijing/501/2009_GQ223411
A/New York/1669/2009_CY039897
A/Beijing/16/2009_HQ698631
A/California/07/2009_FJ969538
A/Texas/45122282/2009_CY052483
A/California/04/2009_FJ966086
A/Taiwan/14/2009_HQ695924
A/Texas/45121004/2009_CY052931
A/California/06/2009_FJ971074
A/Texas/04/2009_FJ981620
A/Texas/08/2009_GQ168853
A/Shanghai/1/2009_GQ225361
A/Qingdao/1170/2009_KF411262
2009 pdmH1N1
A/Boston/21/2009_CY080701
A/Tianjin/15/2009_HQ533873
A/California/VRDL232/2009_CY074279
A/Shanghai/LWS1/2009_GQ850599
A/California/VRDL284/2009_CY074463
A/New York/1185/2009_CY173499
A/Kyoto/08K056/2009_CY043541
A/Taiwan/1026/2009_HQ291859
A/California/VRDL292/2009_CY074487
A/Mexico/24004/2009_CY147591
A/Boston/11/2009_CY080646
A/Boston/1/2009_CY080630
A/Boston/41/2009_CY080797
A/California/VRDL229/2009_CY073865
A/New York/1183/2009_CY173483
2009 seasonal H1N1
0.02

Technical Appendix Figure. Phylogenetic analysis of 12 internal genes of the co-infection viruses in Shanghai, China. Phylogenetic trees were constructed by using the neighbor-joining method with bootstrap analysis of 1,000 replicates in MEGA 5.1 program. Numbers next to nodes indicate bootstrap value percentages (>70%). Panels A, B, C, D, E, F were the PB2, PB1, PA, NP, MP, NS phylogenetic trees of the A/Shanghai/Mix1/2014(H7N9) strain, respectively, and panels G, H, J, K, L, M were the PB2, PB1, PA, NP, MP, NS phylogenetic trees of the A/Shanghai/Mix1/2014(H1N1) strain, respectively. Red font indicates the A/Shanghai/Mix1/2014(H7N9) and A/Shanghai/Mix1/2014(H1N1) strains isolated from the co-infection patient from Shanghai, China. Blue font in the phylogenetic trees of the A/Shanghai/Mix1/2014(H7N9) strain indicates the representative strains of Shanghai area during the same wave of the H7N9 outbreak in China (*1*). Blue font in the phylogenetic trees of the A/Shanghai/Mix1/2014(H1N1) strain indicates the recommended influenza vaccine strain in the 2013–14 Northern Hemisphere influenza season (http://www.who.int/influenza/vaccines/virus/recommendations/2013_14_north/en/). Scale bars indicate nucleotide substitutions per site. The GISAID or GenBank accession numbers of the influenza viruses in the construction of the phylogenetic analyses are displayed in the phylogenetic trees, which followed the taxon names.
